# Dizziness reported by elderly patients in family practice: prevalence, incidence, and clinical characteristics

**DOI:** 10.1186/1471-2296-11-2

**Published:** 2010-01-11

**Authors:** Otto R Maarsingh, Jacquelien Dros, François G Schellevis, Henk C van Weert, Patrick J Bindels, Henriette E van der Horst

**Affiliations:** 1 Department of Family Practice and Institute for Research in Extramural Medicine, VU University Medical Center, Amsterdam, The Netherlands; 2 Department of Family Practice, Academic Medical Center, University of Amsterdam, Amsterdam, The Netherlands; 3 NIVEL, the Netherlands Institute for Health Services Research, Utrecht, The Netherlands; 4 Department of Family Practice, Erasmus University Medical Center, Erasmus University Rotterdam, Rotterdam, The Netherlands

## Abstract

**Background:**

Although dizziness in elderly patients is very common in family practice, most prevalence studies on dizziness are community-based and include a study population that is not representative of family practice. The aim of this study was to investigate the prevalence and incidence of dizziness reported by elderly patients in family practice, to describe their final diagnoses as recorded by the family physician, and to compare the clinical characteristics of dizzy patients with those of non-dizzy patients.

**Methods:**

Data were obtained from the Second Dutch National Survey of General Practice, a prospective registration study which took place over a 12-month period in 2001. We developed a search strategy consisting of 15 truncated search terms (based on Dutch synonyms for dizziness), and identified all patients aged 65 or older who visited their family physician because of dizziness (N = 3,990). We used the mid-time population as denominator to calculate the prevalence and incidence, and for group comparisons we used the Student's t and Chi-square test, and logistic regression analysis.

**Results:**

The one-year prevalence of dizziness in family practice in patients aged 65 or older was 8.3%, it was higher in women than in men, and it increased with age. In patients aged 85 or older the prevalence was similar for men and women. The incidence of dizziness was 47.1 per 1000 person-years. For 39% of the dizzy patients the family physicians did not specify a diagnosis, and recorded a symptom diagnosis as the final diagnosis. Living alone, lower level of education, pre-existing cerebrovascular disease, and pre-existing hypertension were independently associated with dizziness.

**Conclusions:**

Dizziness in family practice patients increases with age. It is more common in women than in men, but this gender difference disappears in the very old. Because a large proportion of dizzy elderly patients in family practice remains undiagnosed, it would be worthwhile to carry out more diagnostic research on dizziness in a family practice setting.

## Background

Dizziness is very common in older people [1-6]. In people aged over 75 it is a frequent reason for visiting a family physician [4]. In many patients dizziness causes serious functional impairment [7-9].

Epidemiological studies focusing on dizziness often have limitations. First of all, dizziness can only be measured by patient self-report, and has a wide range of manifestations. Often it is unclear which manifestations have been included in the definition[10,11]. Another limitation concerns the selection of the study population. Although the majority of dizzy patients are seen in family practice, [12,13] most prevalence studies on dizziness are community-based, and include a spectrum of patients who are not representative of family practice [1-6,14-19]. However, the prevalence of dizziness in the community is likely to be quite different from the prevalence in patients who in fact seek help for this symptom [11].

In the present study we have tried to minimize these limitations, in order to provide representative data on the symptom of dizziness in patients over 65 in family practice. The aim of the study was to investigate the prevalence and incidence of dizziness reported by elderly patients in family practice, to describe their final diagnoses as recorded by the family physician, and to compare the clinical characteristics of dizzy patients with those of non-dizzy patients.

## Methods

In the Netherlands, all non-institutionalized inhabitants are registered with a family physician, an access to a medical specialist is only possible after referral by a family physician. Therefore, morbidity data from family practice provide an accurate reflection of people seeking medical care. The data used in the present study were derived from the Second Dutch National Survey of General Practice (DNSGP-2) [20].

The study was carried out according to Dutch legislation on privacy. The privacy regulation of the study was approved by the Dutch Data Protection Authority. According to the Central Committee on Research Involving Human Subjects (CCMO, http://www.ccmo-online.nl/main.asp?pid=1&taal=1), obtaining informed consent is not obligatory for observational studies.

### Patients and setting

The DNSGP-2 was carried out in 2001 by the Netherlands Institute for Health Services Research (NIVEL) [20]. For this survey, 195 family physicians in 104 practices recorded data about all contacts with their patients for a period of 12 consecutive months. Physicians participating in the DNSGP-2 were partly recruited from the practices participating in the Netherlands Information Network of General Practice, the LINH (http://www.nivel.nl/OC2/page.asp?PageID=8599&path=/Startpunt/NIVEL international/Research/; N = 85 practices). Nineteen practices were recruited on the basis of an additional stratified random sample of practices in the Netherlands. Stratification variables included region, urbanisation level and deprivation area.

The participating family physicians were representative of all family physicians in the Netherlands, with regard to age and gender, and the region and location of the practice. These family physicians had a total practice population of N = 391,294. The study population corresponds very well with the Dutch population with regard to age, gender, and type of health care insurance [20].

The following data were extracted from the DNSGP-2 database: patient characteristics (gender/age/type of health care insurance/level of education), characteristics of consultations (symptom [s] presented/new or existing episode of care/final diagnosis of episode of care), characteristics of prescribed drugs (Anatomical Therapeutic Chemical classification/prescription date/contact diagnosis), and comorbidities. We defined consultation frequency as the number of face-to-face consultations per patient during one year. Polypharmacy was defined as the long-term use of more than five drugs[21]. Long-term use was defined as: 1) three or more prescriptions per drug during a period of one year, and 2) more than 180 days between the first and last date of prescription during the period of one year. Based on the results of previous epidemiological studies on dizziness, we extracted data on the following comorbidities: anxiety syndrome, cataract, cerebrovascular disease, coronary artery disease, depression, diabetes mellitus, hypertension, impaired hearing, impaired vision, and previous myocardial infarction [2,4,6,7,22].

The diagnoses were coded by the family physicians according to the International Classification of Primary Care (ICPC) [23,24]. For each contact they recorded whether it was the first or a subsequent consultation within an episode. If the episode of dizziness included more than one consultation, the diagnosis made during the chronologically last consultation for dizziness was considered to be the final diagnosis of the episode of care.

### Identification of the target population

For the identification of our target population (i.e. patients aged 65 or older who visited their family physician because of a symptom indicating dizziness) we developed a search strategy, because information about the symptoms that were presented was recorded as free-text. The search strategy was based on Dutch synonyms for dizziness, and consisted of fifteen truncated search terms (see Appendix).

We applied the search strategy to the DNSGP-2 database for all patients aged 65 or older. The full-text medical records of identified patients were manually reviewed by a trained medical student, and divided into three subgroups: 1. patients with both dizziness and additional information about the symptom(s) presented, 2. patients with dizziness (recorded ICPC codes A06 'Fainting/syncope', H82 'Vertiginous syndrome', or N17 'Vertigo/dizziness') with no additional information about the symptom(s) presented, and 3. patients without dizziness. A random selection of 5% of the identified medical records was reviewed by a second researcher (OM), to check the reliability of the data-extraction.

The information about the symptom(s) presented was used to assign a subtype of dizziness to each patient: 'vertigo', 'presyncope', 'disequilibrium', or 'no subtype', according to the Drachman and Hart classification [11,25]. Because the family physicians sometimes recorded several symptoms during the same consultation, we occasionally assigned more than one subtype to a patient.

### Data-analysis

The data were analyzed in SPSS version 14.0.2. To determine the one-year prevalence, we calculated the number of patients who consulted their family physician for dizziness at least once during a period of 12 months. To determine the incidence, we calculated the number of patients consulting their family physician for a new episode of dizziness. The incidence rates were calculated per 1000 person-years, grouped according to age, gender, and dizziness subtype. We used the mid-time population of the participating practices as the epidemiological denominator. For the group comparison of men versus women we used a binomial test procedure.

For the group comparison of non-dizzy versus dizzy patients we used the unpaired Student's t test and the Chi-square test, with statistical significance set at p < 0.01. Because of the large sample size, we used a normal approximation to the binomial distribution. We tested the null hypothesis that two proportions were equal for all variables under study. We performed a forward stepwise logistic regression analysis in order to test for independent associations with dizziness. The p-value for entry into the model was set at < 0.05. We calculated the c statistic to determine the discriminative power of the logistic equation. To determine the reliability of our model, we compared the results of the stepwise approach with the results of an "all inclusive" regression analysis.

## Results

### Data-extraction

**Figure 1:**
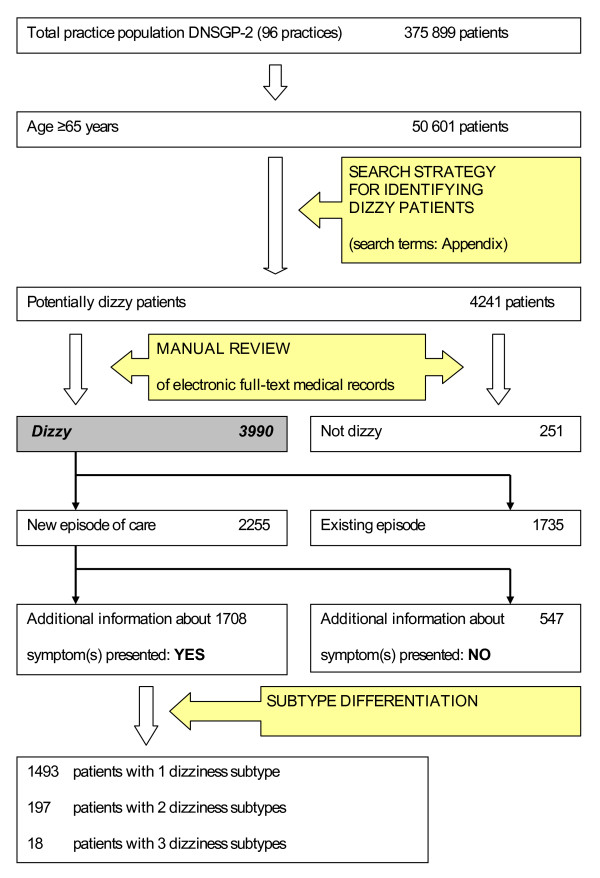
**Flowchart: 3990 dizzy patients aged 65 years or older were identified in the data obtained from the Second Dutch National Survey of General Practice (DNSGP-2)**.

Data from eight practices were excluded because of the poor quality of registration. From the remaining 96 practices we obtained data on 50,601 patients aged 65 or older. By applying our search strategy, we identified 3,990 dizzy patients. These patients had consulted their family physician at least once for dizziness during a period of 12 months (Figure 1). The reliability of the data-extraction was good: from a random selection of 5% of identified potentially dizzy patients, only one out of 213 patients had been classified incorrectly.

### Prevalence

The one-year prevalence of dizziness in family practice in patients aged 65 or older was 83.3 per 1000 persons (Table 1). The prevalence of dizziness in patients aged 65-84 was significantly higher in women than in men (p < 0.001). The prevalence of dizziness increased with age, from 67.8 in the age-group of 65-74 to 108.4 per 1000 persons for patients aged 85 or older.

**Table 1:** One-year prevalence of dizziness in patients aged 65 or older in family practice (per 1000 persons; total practice population DNSGP-2 aged 65 or older: N = 50 601 patients)

	Male (95% CI)	Female (95% CI)	Total (95% CI)
65-74 years	54.4 (51.5-57.3)	79.5 (76.0-82.9)*	67.8 (64.6-71.1)
75-84 years	84.9 (81.3-88.4)	112.1 (108.0-116.1)*	101.6 (97.7-105.4)
≥ 85 years	110.2 (106.2-114.2)	107.7 (103.7-111.7)	108.4 (104.4-112.4)
Total	67.6 (64.4-70.9)	94.6 (90.9-98.4)*	83.3 (79.8-86.8)

*: statistically significant difference between men and women (p < 0.001)

DNSGP-2: the Second Dutch National Survey of General Practice; CI: confidence interval

### Incidence

During the registration year 2,255 dizzy patients consulted their family physician for a new episode of care. The incidence of dizziness was 47.1 per 1000 person-years. The medical records of 1,708 incident patients (75.7%) contained additional information about the symptom(s) presented. Based on this information we could assign one dizziness subtype to 1,493 patients, two subtypes to 197 patients, and three subtypes to 18 patients (Figure 1).

The incidence rates of all dizziness subtypes except 'vertigo' increased with age (Table 2). The incidence of dizziness in patients aged 65-84 was significantly higher in women than in men (p < 0.001). For the groups with a known specified subtype, the incidence of 'vertigo' was significantly higher in women than in men (p < 0.001), whereas the incidence of 'presyncope' and 'disequilibrium' was similar for men and women in all age groups.

**Table 2:** Incidence of different subtypes of dizziness in patients aged 65 or older in family practice (per 1000 person-years; total practice population DNSGP-2 aged 65 or older: N = 50 601 patients)

		Vertigo	Presyncope	Disequilibrium	No subtype	Subtype unknown	Total
65-74 years	Male (95% CI)	5.3 (4.4-6.2)	6.6 (5.5-7.6)	2.3 (1.7-2.9)	14.9 (13.3-16.4)	7.5 (6.4-8.6)	36.6 (34.2-39.0)
	Female (95% CI)	7.4 (6.3-8.5)*	6.7 (5.6-7.7)	2.6 (2.0-3.3)	23.5 (21.6-25.5)*	10.6 (9.3-11.9)*	50.8 (48.0-53.6)*
	Total (95% CI)	6.4 (5.3-7.4)	6.6 (5.6-7.7)	2.5 (1.9-3.1)	19.5 (17.7-21.3)	9.2 (8.0-10.4)	44.2 (41.5-46.8)
							
75-84 years	Male (95% CI)	3.8 (3.0-4.6)	9.0 (7.8-10.3)	3.5 (2.7-4.3)	23.2 (21.2-25.1)	13.2 (11.7-14.6)	52.7 (49.8-55.5)
	Female (95% CI)	7.1 (6.0-8.1)*	10.0 (8.8-11.3)	4.4 (3.5-5.2)	29.9 (27.7-32.1)*	13.9 (12.4-15.4)	65.3 (62.2-68.5)*
	Total (95% CI)	5.8 (4.8-6.8)	9.7 (8.4-10.9)	4.0 (3.2-4.9)	27.3 (25.2-29.4)	13.6 (12.1-15.1)	60.4 (57.4-63.5)
							
≥ 85 years	Male (95% CI)	3.8 (3.0-4.5)	12.0 (10.6-13.4)	8.3 (7.1-9.4)	30.0 (27.8-32.2)	15.0 (13.4-16.6)	69.0 (65.8-72.2)
	Female (95% CI)	4.8 (4.0-5.7)	12.1 (10.7-13.5)	5.1 (4.2-6.1)	26.9 (24.9-29.0)	17.2 (15.6-18.9)	66.3 (63.7-69.4)
	Total (95% CI)	4.5 (3.7-5.4)	12.1 (10.7-13.5)	6.0 (5.0-7.0)	27.8 (25.7-29.9)	16.6 (15.0-18.2)	67.0 (63.8-70.2)
							
Total	Male (95% CI)	4.7 (3.8-5.5)	7.7 (6.6-8.8)	3.1 (2.4-3.8)	18.5 (16.8-20.2)	9.8 (8.5-11.0)	43.7 (41.1-46.3)
	Female (95% CI)	7.0 (5.9-8.0)*	8.5 (7.4-9.7)	3.6 (2.8-4.3)	26.3 (24.2-28.3)*	12.6 (11.2-14.0)*	57.9 (54.9-60.9)*
	Total (95% CI)	6.0 (5.0-7.0)	8.2 (7.0-9.3)	3.4 (2.6-4.1)	23.0 (21.1-24.9)	11.4 (10.1-12.8)	51.9 (49.1-54.8)

*: statistically significant difference between men and women (p < 0.001)

DNSGP-2: the Second National Survey of General Practice; CI: confidence interval

### Final diagnoses

The family physicians recorded one final diagnosis for 1,660 patients (97.2%), two final diagnoses for 47 patients (2.8%), and three final diagnoses for one patient (0.1%). They often recorded a symptom diagnosis as final diagnosis (39.0%, Table 3). The most frequently recorded diagnoses were vertigo/dizziness (28.0%), vertiginous syndrome (11.9%, including Benign Paroxysmal Positional Vertigo, labyrinthitis, Ménière's disease, and vestibular neuronitis), and fainting/syncope (8.5%).

**Table 3:** Frequency of final diagnoses as recorded by the family physician during one year of registration in 1708 elderly patients with a new episode of dizziness

Diagnoses	N	%*
I. Symptom diagnoses (listed if > 1% of total)	666	39.0
N17 Vertigo/dizziness (excl. H82)	478	28.0
A06 Fainting/syncope	146	8.5
A04 General weakness/tiredness	42	2.5
II. Cardiovascular conditions	245	14.3
K89 Transient cerebral ischemia	35	2.0
K88 Postural hypotension	33	1.9
K90 Stroke/cerebrovascular accident	27	1.6
K86 Hypertension uncomplicated	26	1.5
K78 Atrial fibrillation/flutter	16	0.9
Other	108	6.3
III. Peripheral vestibular disease	203	11.9
H82 Vertiginous syndrome†	203	11.9
IV. Psychiatric conditions	97	5.7
P01 Feeling anxious/nervous/tense	24	1.4
A-Z26/A-Z27 Fear of disease	14	0.8
R98 Hyperventilation syndrome	12	0.7
P76 Depressive disorder	11	0.6
Other	36	2.1
V. Musculoskeletal conditions	90	5.3
VI. Infection	69	4.0
VII. Adverse effect medical agent	51	3.0
VIII. Metabolic or endocrine conditions	30	1.8
IX. Neurologic conditions (excluding cerebrovascular conditions)	26	1.5
Other	280	16.4
		
Total	1757	102.9

*: Adds up to more than 100%, because family physicians recorded one final diagnosis for 1660 patients, two final diagnoses for 47 patients, and three final diagnoses for 1 patient (total of 1757 diagnoses)

†: Including Benign Paroxysmal Positional Vertigo, labyrinthitis, Ménière's disease, and vestibular neuronitis

### Dizzy versus non-dizzy patients

Univariate analysis showed that dizzy patients were significantly older (76.1 vs. 74.5 years, Table 4), were more often female (65.9 vs. 57.2%), were more often living alone (34.8 vs. 25.6%), more often had public health care insurance (77.3 vs. 72.8%), and more often had a significantly lower level of education (elementary school: 43.6 vs. 37.4%). Compared to non-dizzy patients, dizzy patients visited their family physician significantly more often (12.8 vs. 6.3 consultations in one year), took more long-term drugs (2.3 vs. 1.6), had higher rates of polypharmacy (11.0 vs. 6.3%), and had higher rates of pre-existing comorbidities. The factors education and medical history had a high percentage of missing values (22 and 23%).

**Table 4:** Socio-demographic characteristics, consultation frequency, long-term drug use and medical history in dizzy and non-dizzy patients aged 65 years or older in family practice

	Prevalence (%)			
			
	Non-dizzy (n = 46611)	Dizzy (n = 3990)	Univariate p	Multivariate* OR (95% CI)
Gender, female	57.2	65.9	< 0.001†	
Age in years, mean (SEM)	74.5 (0.03)	76.1 (0.11)	< 0.001†	
Health care insurance			< 0.001†	
Public health	72.8	77.2		
Private	27.2	22.7		
Unknown/missing	0.1	0.1		
Living alone			< 0.001†	1.3 (1.2-1.4)
Yes	25.6	34.8		
No	69.8	60.6		
Unknown/missing	4.6	4.6		
Education			< 0.001†‡	
None	1.4	1.5		
Elementary school	37.4	43.6		1.2 (1.1-1.3) §
High school	32.5	30.2		
College or university	6.4	4.2		
Unknown/missing	22.4	20.5		
Consultation frequency, mean (SEM)	6.3 (0.03)	12.8 (0.18)	< 0.001†	
Long-term drug use||				
Number of drugs, mean (SEM)	1.6 (0.01)	2.3 (0.04)	< 0.001†	
Polypharmacy (> 5 drugs)	6.3	11.0	< 0.001†	
Medical history				
Number of diagnoses, mean (SEM)	1.9 (0.02)	2.5 (0.06)	< 0.001†	
Anxiety syndrome	0.2	0.5	< 0.001†	
Cataract	2.0	3.7	< 0.001†	
Cerebrovascular disease	2.9	5.3	< 0.001†	1.3 (1.1-1.5)
Coronary artery disease	7.5	11.4	< 0.001†	
Depression	1.7	3.0	< 0.001†	
Diabetes mellitus	7.3	9.8	< 0.001†	
Hypertension	12.8	18.1	< 0.001†	1.2 (1.1-1.3)
Impaired hearing	2.7	4.2	< 0.001†	
Impaired vision	0.6	1.0	0.006†	
Myocardial infarction	2.1	2.8	0.02	
Unknown/missing	24.1	17.4		

*: Forward stepwise logistic regression (entry p < 0.05, removal p < 0.1), adjusted for gender, age and consultation frequency

†: Statistically significant difference between non-dizzy and dizzy patients ≥ 65 years (p < 0.01)

‡: Linear-by-Linear Association

§ Reference: college or university

||: Criteria: a) ≥ 3 prescriptions per drug during one year of registration

b) > 180 days between first and last date of prescription per drug during one year of registration

OR = odds ratio; CI = confidence interval; SEM = standard error of the mean

In multivariate analysis (adjusted for gender, age and consultation frequency) four factors were independently associated with dizziness: living alone (odds ratio [OR] 1.3; 95% confidence interval 1.2-1.4), a lower level of education (elementary school compared to college/university, OR 1.2 [1.1-1.3]), pre-existing cerebrovascular disease (OR 1.3 [1.1-1.5]), and pre-existing hypertension (OR 1.2 [1.1-1.3]). The calculated c statistic was 0.73 (satisfactory discriminative power). The results of the forward stepwise logistic regression analysis and the "all inclusive" regression analysis did not differ.

## Discussion

### Summary of main findings

In this study, the one-year prevalence of dizziness in family practice in patients aged 65 or older was 8.3%. In general, the prevalence was higher in women than in men, and increased with age. However, the prevalence in the very old (≥ 85 years) was similar for men and women. The incidence of dizziness in family practice was 47.1 per 1000 person-years. The incidence rates of all subtypes except 'vertigo' increased with age. The incidence rate for the subtype 'vertigo' was higher in women than in men. The incidence rates for the subtypes 'presyncope' and 'disequilibrium' were similar for men and women in all age-groups. For about 40% of the patients the family physicians did not specify a diagnosis, and recorded a symptom diagnosis as the final diagnosis. Living alone, a lower level of education, pre-existing cerebrovascular disease, and pre-existing hypertension were independently associated with dizziness.

### Strengths and limitations of this study

Although the majority of dizzy patients are seen in family practice, [12,13] most prevalence studies on dizziness are community-based, and include a study population that is not representative of family practice. The present study is representative of family practice, has a large sample size, and uses the symptom(s) presented by the patient as a starting point.

A limitation of our study is its dependence on the quality of registration by the family physicians. It is possible that some family physicians incorrectly recorded a subsequent consultation as the first consultation for dizziness. This could have caused an overestimation of the incidence rates of dizziness. However, we consider such an overestimation to be limited, because all of the family physicians were trained to record episodes of care, and all episodes that were classified as a 'new episode of care' were checked twice for incorrect classification, both during the DNSGP-2 data-collection, [20] and during the present study. For one fourth of patients with a new episode of care the family physicians did not record the symptom(s) presented, but only an ICPC-based code for dizziness, so for this group of patients assignment to a dizziness subtype was not possible. Although this does not affect the prevalence rates, it causes an underestimation of the incidence rates for the different dizziness subtypes. It also implies a risk of selection bias: it is imaginable that some family physicians failed to record the symptom(s) presented by certain patients (for example patients with common, benign causes of dizziness). This can cause an underestimation of the contribution of this group of diagnoses to the subtypes of dizziness (Table 2).

Furthermore, we emphasize that Table 3 describes the diagnoses routinely recorded by the family physicians. However, it is not the yield of a standardized prospective diagnostic study.

The comparison of non-dizzy with dizzy patients (Table 4) also has some limitations. Firstly, although many factors are plausible, and have been found to be associated with dizziness in previous studies, we cannot determine a causal relationship because of the cross-sectional design of the study. Secondly, for some factors the percentage of missing values is high, especially with regard to level of education and medical history. Although the multivariate analysis showed no independent association for these missing values, a disturbing effect is possible. Thirdly, our definition of long-term drug use is merely an attempt to compensate for missing information about the duration of a prescription. However, the results are comparable to those of a Dutch polypharmacy study in family practice[21]. Finally, the list of potential factors is not exhaustive, but a selection based on previous studies [2,4,6,7,22].

### Comparison with existing literature

Compared to the results of another prevalence study on dizziness representative of primary care, [13] the prevalence rates we found were almost twice as high for all studied age-groups. This may be due to the studied population, because Sloane et al. included patients of family physicians, general practitioners, general internists, and general paediatricians. Kruschinski et al. also reported a lower prevalence[26]. However, this may be due to the younger age of their study population (mean age 59 years), a different classification system (ICD-10 vs. ICPC), and a different method of data retrieval. In a longitudinal population-based study among people above 65 years, 11% of the participants reported dizziness problems, which is consistent with our study[27].

Previous prevalence studies carried out in a community-based population have reported much higher prevalence rates (15-50%) [1-6,14-19]. This is probably due to the fact that complaints of dizziness do not automatically lead to a medical consultation[16].

Contrary to the findings of other studies, [1-3,10,11,13] we found no gender differences with regard to prevalence and incidence rates in the oldest patients. This may be due to the fact that the relative contribution of gender-specific diagnoses, such as vestibular vertigo which is much more common in women, [16] decreases with age.

There are no previous studies on dizziness that have investigated the incidence of subtypes of dizziness in different age-groups. Our finding that the incidence rates of all dizziness subtypes increased with age, except for the subtype 'vertigo', may be due to the fact that the relative contribution of 'non-vestibular' causes of dizziness (such as cardiovascular conditions) increases with age.

In a community-based study, Neuhauser et al. reported an annual incidence of "dizziness/vertigo leading to a medical consultation" of 1.8%, [16] which may seem low compared to our study (annual incidence of 5%). However, this may be due to the younger age of the studied population (18-79 years), and a different research method (survey).

The family physicians recorded a symptom diagnosis as final diagnosis for 39% of the dizzy patients, i.e. no diagnosis could be made after opportunities for further confirmation (such as follow-up consultations, additional diagnostic tests, or a referral). Previous studies that have investigated causes of dizziness in primary care have reported varying percentages of dizziness with unknown cause, ranging from 0-5% [28,29] to 22-37% [30-32].

Contrary to the findings of previous studies, [6,22] in the present study living alone was found to be associated with dizziness. This association might be due to the fact that people who live alone are more likely to report dizziness, for example because they have fewer people to reassure them. An inverse association with level of education has been found in earlier studies, not only for patients with vestibular vertigo[33], but also for various health conditions that are not related to dizziness[34]. The factors pre-existing cerebrovascular disease and hypertension have been investigated in several previous studies, but only reported to be associated with dizziness by Sloane et al [2,4,6,22]. Previously reported associations with cataract, [22] diabetes, [2,22] impaired hearing, [6] previous myocardial infarction, [2,6,22] polypharmacy, [6,22] and psychiatric comorbidity could not be confirmed [4,6,7,22]. However, these associations may be absent in our study because of the high percentage of missing values for the factor medical history.

### Implications for future research

It would be worthwhile to perform a prospective cohort study that uses Drachman's classification as a starting point, [25] because the present study does not provide complete information about the incidence of each subtype of dizziness. Furthermore, the absence of gender differences in the incidence rates of the dizziness subtypes 'presyncope' and 'disequilibrium' needs to be confirmed in a new study. Finally, given the large proportion of undiagnosed dizzy patients in family practice, it would be worthwhile to carry out more diagnostic research on dizziness in a family practice setting. Although an increase in specific diagnoses does not necessarily imply an increase in specific therapies, such research may provide more 'diagnostic tools' for family physicians in daily clinical practice.

## Conclusions

In this registration study with a large and representative sample, we have used the symptom(s) presented by the patient as a starting point.

Dizziness in patients in family practice increases with age. It is more common in women than in men, but this gender difference disappears in the very old. Because a large proportion of dizzy elderly patients in family practice remains undiagnosed, it would be worthwhile to carry out more diagnostic research on dizziness in a family practice setting.

## Abbreviations

CI: confidence interval; DNSGP-2: the Second Dutch National Survey of General Practice; ICPC: International Classification of Primary Care; NIVEL: the Netherlands Institute for Health Services Research; OR: odds ratio; SEM: standard error of the mean.

## Competing interests

The authors declare that they have no competing interests.

## Authors' contributions

FS designed the DNSGP-2. HvdH and HvW designed the present study and obtained the funding. OM extracted the data, performed the statistical analyses with FS, and wrote the original draft. OM, JD, FS, HvW, PB and HvdH revised the draft critically with regard to important intellectual content, and approved the final version of the paper.

## Appendix

Search terms for identifying patients with symptoms related to dizziness

**Table:** 

*Search term -*	*Symptom in Dutch -*	*English translation*
1. draai*	draaierig	giddy/spinning sensation (V)
2. vertig*	vertigo	vertigo (V)
3. zweve*	zweverig	giddy (V)
4. collab*	collaberen	collapsing (P)
5. collap*	collaps	collapse (P)
6. flauw*	flauwte	faint feeling (P)
7. licht in	licht in het hoofd	lightheadedness (P)
8. onwel*	onwelwording	becoming unwell (P)
9. zwart voor	zwart voor de ogen	everything turning black (P)
10. evenwicht*	evenwichtsstoornis	loss of equilibrium (E)
11. onvast*	onvast (ter been)	instability (E)
12. valnei*	valneiging	tendency to fall (E)
13. wankel*	wankel (ter been)	to be unsteady on one's legs (E)
14. dizz*	dizzy	dizzy (N)
15. duizel*	duizeligheid	dizziness (N)

*: truncation

V: subtype vertigo; P: subtype presyncope; D: subtype disequilibrium; N: no subtype[11,25].

## Pre-publication history

The pre-publication history for this paper can be accessed here:

http://www.biomedcentral.com/1471-2296/11/2/prepub
